# Long-term glycemic variability and the risk of heart failure: a meta-analysis

**DOI:** 10.7717/peerj.20401

**Published:** 2025-11-27

**Authors:** Yong-Chao Li, Ke-Er Mo, Li-Shuai Zhang, Qiang Zhao, Ju Deng, Li Li

**Affiliations:** 1 Jinan University, Guangzhou, China; 2 Department of Critical Care Rehabilitation, The First People’s Hospital of Chenzhou, Chenzhou, China; 3 Department of Cardiology, Guangzhou Red Cross Hospital, Guangzhou, China

**Keywords:** Heart failure, Glycemic variability, Risk factor, Hyperglycemia, Meta-analysis

## Abstract

**Background:**

Long-term glycemic variability (GV) has emerged as a potential cardiovascular risk factor beyond average glycemic levels. However, its association with the risk of heart failure (HF) remains unclear. This meta-analysis evaluated the relationship between long-term GV and the incidence of HF in adults.

**Methods:**

We systematically searched PubMed, Embase, and Web of Science from inception to January 31, 2025, for observational studies assessing the association between long-term GV—measured by variability indices of hemoglobin A1c (HbA1c) or fasting plasma glucose—and HF risk. Pooled hazard ratios (HRs) with 95% confidence intervals (CIs) were calculated using random-effects models by incorporating the influence of heterogeneity.

**Results:**

Eleven datasets from 10 studies involving 4,229,377 adults were included. Compared with participants with low GV, those with high long-term GV had a significantly increased risk of incident HF (HR = 1.69; 95% CI [1.38–2.06]; *p* < 0.001; I^2^ = 92%). The association remained consistent in sensitivity analyses restricted to patients with type 2 diabetes, high-quality studies, and studies adjusting for mean hemoglobin A1c (HbA1c) levels (HR = 1.96, 1.78, and 1.95, respectively; all *p* < 0.001). Subgroup analyses revealed consistent findings across GV metrics, geographic regions, study designs, mean age, sex distribution, follow-up duration, and study quality (*p* for subgroup difference > 0.05). No significant publication bias was detected (Egger’s test, *p* = 0.29).

**Conclusion:**

High long-term GV is independently associated with an increased risk of HF. These findings underscore the clinical relevance of GV monitoring in cardiovascular risk assessment, including risk stratification for the incidence of HF.

## Introduction

Heart failure (HF) is a major global public health concern, affecting an estimated 64 million people globally (Martin et al., 2025; Savarese et al., 2023). Its burden is increasing with aging in populations and the rising prevalence of cardiovascular risk factors (Shahim et al., 2023). HF is associated with high morbidity, frequent hospitalization, and substantial mortality, with 5-year survival rates comparable to those of many cancers (Mamas et al., 2017). In addition to its effects on individuals, HF imposes a significant economic burden on healthcare systems associated with chronic disease management and recurrent admissions (Urbich et al., 2020). Although established risk factors such as hypertension, coronary artery disease, diabetes mellitus, obesity, and aging are well recognized, the prognosis of HF remains poor, and identifying novel, modifiable risk factors is crucial for early prevention and improved outcomes (Bui, Horwich & Fonarow, 2011; Lüscher, 2015; Meijers & de Boer, 2019).

Glycemic control has long been a cornerstone of diabetes management, with hemoglobin A1c (HbA1c) serving as a widely accepted marker of average glucose levels (Saudek & Brick, 2009). However, emerging evidence suggests that fluctuations in glycemic levels over time—known as glycemic variability (GV)—might have independent prognostic value beyond mean glycemia (Suh & Kim, 2015). GV can be broadly categorized as short-term (within-day or between-day fluctuations, typically assessed *via* continuous glucose monitoring (CGM)) or long-term (visit-to-visit fluctuations over months or years, often assessed using HbA1c or fasting plasma glucose (FPG) records) (Suh & Kim, 2015; Zhou et al., 2020). Long-term GV is commonly measured using indices such as standard deviation (SD), coefficient of variation (CV), variability independent of the mean (VIM), and average successive variability (ASV) (Zhou et al., 2020). Unlike transient glycemic excursions, long-term GV might reflect a sustained pattern of metabolic instability, potentially leading to cumulative damage to target organs (Sun, Luo & Zhou, 2021).

There is growing interest in the potential link between long-term GV and cardiovascular disease (CVD), including myocardial infarction, stroke, and cardiovascular mortality (Chen et al., 2022; Gorst et al., 2015). Several pathophysiological mechanisms could explain this relationship, such as oxidative stress, inflammation, endothelial dysfunction, and autonomic imbalance induced by repeated glucose fluctuations (Helleputte et al., 2020; Papachristoforou et al., 2020). These effects can promote myocardial remodeling, fibrosis, and neurohormonal activation, thereby contributing to the development of HF (Elendu et al., 2023). However, despite increasing research, the association between long-term GV and the risk of HF remains uncertain. Whereas some cohort studies demonstrated a significant association (Ceriello et al., 2022; Gu et al., 2018; Kwon et al., 2019; Li et al., 2020; Manosroi et al., 2023; Segar et al., 2020; Wan et al., 2020; Wang et al., 2023), others reported null findings (Kaze et al., 2020; Lin et al., 2021). In view of this uncertainty, we conducted a systematic review and meta-analysis to comprehensively evaluate the association between long-term GV and the risk of incident HF in adult populations.

## Materials and Methods

This meta-analysis followed the PRISMA 2020 guidelines (Higgins et al., 2021; Page et al., 2021) and the Cochrane Handbook for Systematic Reviews and Meta-Analyses (Higgins et al., 2021) for protocol design, data extraction, statistical analysis, and results reporting. The study protocol was also registered in PROSPERO under ID CRD420251000563.

### Literature search

Relevant studies for this meta-analysis were identified through a comprehensive search in PubMed, Embase, and Web of Science using a broad range of search terms as follows: (1) “glycosylated hemoglobin” OR “HbA1c” OR “glucose” OR “glycemic;” (2) “variability” OR “variation” OR “fluctuation” OR “coefficient of variation” OR “standard variation;” (3) “heart failure” OR “cardiac failure” OR “cardiac dysfunction;” and (4) “incidence” OR “risk” OR “cohort” OR “longitudinal” OR “prospective” OR “retrospective” OR “prospectively” OR “retrospectively” OR “followed” OR “follow-up”. The search was limited to human studies and full-length articles published in English in peer-reviewed journals. Additionally, references from relevant original and review articles were manually screened to identify additional eligible studies. The search spanned from database inception to January 31, 2025.

### Inclusion and exclusion criteria

The eligibility criteria for studies were established using the PICOS framework.

P (patients): General adult population (≥18 years), regardless of the diabetic status of the participants.

I (exposure): The exposure of interest in this meta-analysis was long-term GV, specifically measured by HbA1c or FPG variability indices such as SD, CV, VIM, and ASV. Only studies that quantified HbA1c or FPG variability over an extended period (*e.g.*, months to years) and reported its association with the risk of HF were included. The cutoffs for defining a high long-term GV were consistent with the values used in the original studies.

C (comparison): Participants with low long-term GV at baseline.

O (outcome): Incidence of HF, compared between participants with high long-term GV *versus* those with low long-term GV at baseline.

S (study design): Observational studies with longitudinal follow-up, such as cohort studies, nested case-control studies, or *post-hoc* analyses of clinical trials.

Reviews, editorials, meta-analyses, preclinical research, those including pediatric patients, those not using long-term GV as an exposure, and those that did not report the incidence of HF were excluded. Studies assessing short-term glycemic fluctuations, such as daily glucose variability or CGM metrics, were also excluded. When population overlap occurred, the study with the largest sample size was selected for inclusion in the meta-analysis.

### Study quality assessment and data extraction

Two authors (Yong-Chao Li and Ke-Er Mo) independently conducted the literature search, study selection, quality assessment, and data extraction. Any disagreements regarding study selection or data extraction were resolved through discussion. If consensus could not be reached, the final decision was made by the corresponding author, Li Li, who acted as the adjudicating referee. Study quality was evaluated using the Newcastle–Ottawa Scale (NOS) (Wells et al., 2010), which assesses selection, confounding control, and outcome measurement. NOS scores range from 1 to 9, with nine denoting the highest quality. Studies with NOS scores of seven higher are considered high-quality studies. The data extracted for analysis included study characteristics (author, year, country, and design), participant characteristics (source of the population, age, sex, and diabetic status), exposure characteristics (parameters, cutoffs, and times of glycemic parameters used for GV calculation), follow-up durations, outcome characteristics (definition of HF outcome and the number of patients with newly developed HF), and variables adjusted or matched in estimating the relationship between long-term GV and the risk of HF.

### Statistical analyses

The association between long-term GV and HF risk in adults was presented as hazard ratios (HRs) and the corresponding 95% confidence intervals (CIs) between participants with high long-term GV and those with low long-term GV at baseline. If a study reported multiple GV indices based on the same population, only the HR associated with the largest effect size (*i.e.,* highest HR) was included to avoid data duplication and maintain statistical independence (Higgins et al., 2021). HRs and their standard errors were calculated from 95% CIs or *p*-values and log-transformed to stabilize variance and normalize the distribution (Higgins et al., 2021). To assess heterogeneity, we used the Cochrane Q test and *I*^2^ statistic (Higgins & Thompson, 2002), with *I*^2^ < 25%, *I*^2^ = 25–75%, and *I*^2^ > 75% indicating mild, moderate, and substantial heterogeneity among the included studies, respectively. A random-effects model was used to synthesize results while accounting for variability across the studies (Higgins et al., 2021). Sensitivity analysis was conducted by sequentially excluding individual studies to assess the robustness of the findings (Marušić et al., 2020). In addition, predefined subgroup analyses were also performed to evaluate the effects of study characteristics, such as different GV parameters, study countries (Asian *versus* Western), study design (prospective *versus* retrospective and *post-hoc* analyses), mean participant ages, proportion of men, mean follow-up durations, and NOS scores, on the results. Subgroups were defined using the median values of continuous variables as cutoffs. Publication bias was assessed through funnel plots, visual asymmetry inspection, and Egger’s regression test (Egger et al., 1997). *p* < 0.05 indicated statistical significance. Statistical analyses were conducted using RevMan (Version 5.1; Cochrane Collaboration, Oxford, UK) and Stata software (version 12.0; Stata Corporation, College Station, TX, USA).

## Results

### Study identification

**Figure 1:**
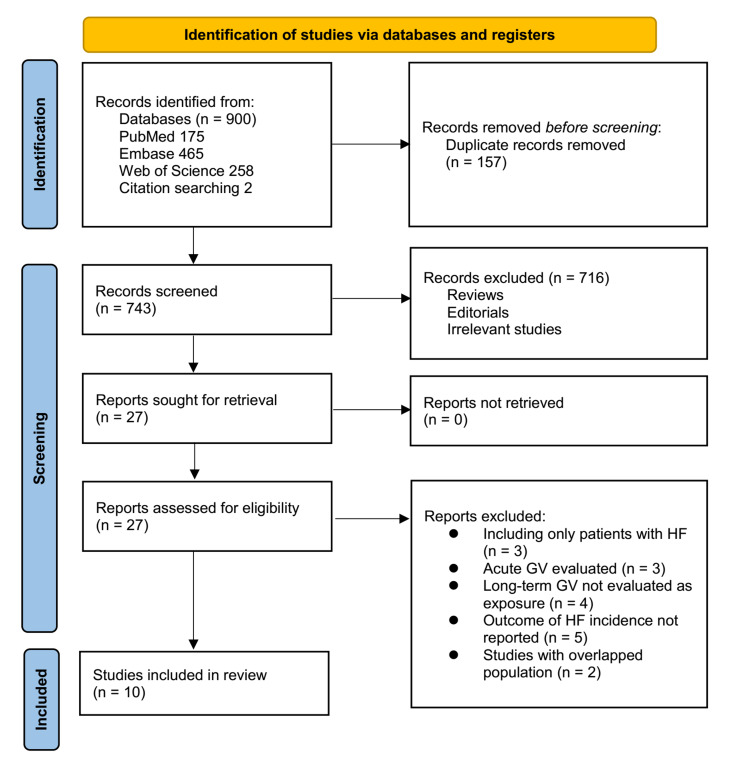
Flowchart of the database search and study inclusion.

Figure 1 outlines the study selection process. Initially, 900 records were identified across three databases, and 157 duplicates were removed. After title and abstract screening, 716 articles were excluded for failing to meet the meta-analysis criteria. The full texts of the remaining 27 studies were independently reviewed by two authors, leading to the exclusion of 17 for reasons detailed in Fig. 1. Ultimately, 10 studies were included in the quantitative analysis (Ceriello et al., 2022; Gu et al., 2018; Kaze et al., 2020; Kwon et al., 2019; Li et al., 2020; Lin et al., 2021; Manosroi et al., 2023; Segar et al., 2020; Wan et al., 2020; Wang et al., 2023).

### Overview of the study characteristics

Table 1 presents a summary of the characteristics of the studies included in the meta-analysis. Because one study (Wang et al., 2023) included two cohorts, these datasets were independently included in the meta-analysis, making 11 datasets available. Overall, three prospective cohorts (Manosroi et al., 2023; Wan et al., 2020; Wang et al., 2023), six retrospective cohorts (Ceriello et al., 2022; Gu et al., 2018; Kwon et al., 2019; Li et al., 2020; Lin et al., 2021; Wang et al., 2023), and two *post-hoc* analyses of clinical studies (Kaze et al., 2020; Segar et al., 2020) were included. These studies were published from 2018 to 2023, and they were conducted in China, Korea, the United States, the United Kingdom, Hong Kong (China), Taiwan (China), Sweden, and Thailand. Community adults were included in three cohorts (Kwon et al., 2019; Wang et al., 2023), adults with type 2 diabetes mellitus (T2DM) were included in seven cohorts (Ceriello et al., 2022; Gu et al., 2018; Kaze et al., 2020; Li et al., 2020; Lin et al., 2021; Segar et al., 2020; Wan et al., 2020), and another cohort included people with prediabetes or T2DM (Manosroi et al., 2023). Overall, 4,229,377 adults were included, with the mean ages of the participants varying from 51.6 to 65.3 years and the proportion of men varying from 37.9% to 78.8%. Multiple parameters were used in these studies to evaluate long-term GV, such as HbA1c-SD, HbA1c-CV, HbA1c-ASV, HbA1c-VIM, FPG-SD, FPG-CV, FPG-ASV, FPG-VIM, and the HbA1c variability score. The cutoffs for defining high long-term GV also varied, with studies using medians (Gu et al., 2018), tertiles (Lin et al., 2021), quartiles (Ceriello et al., 2022; Kaze et al., 2020; Kwon et al., 2019; Manosroi et al., 2023; Wang et al., 2023), quintiles (Segar et al., 2020), and predefined cutoffs of the GV parameters (Li et al., 2020; Wan et al., 2020). The mean follow-up duration ranged from 4.4–16.2 years. HF was diagnosed by clinical evaluation according to international HF guidelines in two cohorts (Gu et al., 2018; Wang et al., 2023), HF hospitalization in one cohort (Manosroi et al., 2023), first HF hospitalization or HF-related death in three cohorts (Kaze et al., 2020; Li et al., 2020; Segar et al., 2020), and ICD codes indicating HF in five cohorts (Ceriello et al., 2022; Kwon et al., 2019; Lin et al., 2021; Wan et al., 2020; Wang et al., 2023). Accordingly, 32,195 (0.76%) patients had newly developed HF during follow-up. Multivariate analyses were performed in all studies when the association between long-term GV and the risk of HF was estimated, with adjustments performed for age, sex, diabetic status, comorbidities, and concurrent cardiovascular medications to varying degrees. NOS scores ranged from 6–9, indicating moderate-to-high methodological and reporting quality Table 2.

**Table 1:** Characteristics of the included studies.

Study	Country	Design	Participant characteristics	Sample size	Mean age (years)	Male sex (%)	DM (%)	Parameters for GV	Cutoffs for GV parameters	Times glycemic parameters used for GV calculation	Mean follow-up duration (years)	Definition of HF outcome	No. of patients with HF	Variables adjusted or matched
Gu 2018	China	RC	T2DM and HTN without clinical signs or symptoms of HF	201	65.3	60.2	100.0	HbA1c-SD and HbA1c-CV	Median	HbA1c measurement: 11.8 times (mean)	7.3	Symptomatic HFpEF as clinically diagnosed according to the 2013ACCF/AHA guideline of HF	18	Age, sex, SBP, DBP, HbA1c-mean, eGFR, BMI, duration of T2DM and hypertension, AF, medical treatment, LAD, LVMI, E/E0, and LVEF
Kwon 2019	Korea	RC	General population aged ≥40 years who had undergone ≥3 health check-ups	3,820,191	51.6	52.9	0.0	FPG-VIM, FPG-SD, and FPG-CV	Q4:Q1	FPG measurement: at least three times	5.3	ICD codes evidenced HF diagnosed in outpatient clinic or during hospitalization	17,253	Age, sex, smoking status, alcohol consumption, exercise, and income, baseline BP, BMI, FBG, TC, histories of IHD, CKD, and COPD
Kaze 2020	USA	*Post-hoc* analysis	Overweight or obese adults with T2DM aged 45–76 years	3,560	58.4	37.9	100.0	HbA1c-SD, HbA1c-CV, HbA1c-ASV, HbA1c-VIM, FPG-SD, FPG-CV, FPG-ASV, and FPG-VIM	Q4:Q1	HbA1c and FPG measurement: four times	6.8	First HF hospitalization or HF-related death	91	Age, sex, race/ethnicity, randomization arm, BMI, current smoking, alcohol drinking, use of BP-lowering medication, average total cholesterol-to-HDL cholesterol ratio, eGFR, duration of diabetes, average SBP, and average HbA1c
Segar 2020	USA	*Post-hoc* analysis	Patients with T2DM	8,576	62.4	61.6	100.0	HbA1c-ASV	Q5:Q1	HbA1c measurement: eight times(median); FPG measurement: four times (median)	6.4	First HF hospitalization or HF-related death	388	Age, sex, race, level of education, randomization arm, history of CVD, conventional CV risk factors, CV medications, and baseline HbA1c
Li 2020	UK	RC	Patients with T2DM aged ≥40 years	19,059	63.3	54.6	100.0	HbA1c variability score	>80 versus <20	HbA1c measurement: 12 times (median)	6.8	First HF hospitalization or HF-related death	853	Age, sex, smoking, hypertension, BMI, HDL cholesterol, eGFR, antiplatelet therapy, and CCI
Wan 2020	Hongkong (China)	PC	Patients with T2DM aged 45–84 years	147,811	64.2	46	100.0	HbA1c-SD	≥3.0 versus <0.24	HbA1c measurement: 3.2 times (median)	7.4	ICD codes evidenced HF	7,908	Age, sex, smoking, duration of diabetes, BMI, SBP, DBP, LDL cholesterol, eGFR, antidiabetics, lipid lowering agents, CCI, and usual HbA1c
Lin 2021	Taiwan (China)	RC	Patients with T2DM	3,824	57.8	50.2	100.0	HbA1c-SD	T3:T1	HbA1c measurement: at least three times	11.7	ICD codes evidenced HF	315	Age, sex, diabetes duration, BMI, SBP, TC, TG, HDL-C, LDL-C, eGFR, CAD, HTN, stroke, antidiabetics, and CV medications
Ceriello 2022	Sweden	RC	Patients with T2DM	101,533	64.3	55.6	100.0	HbA1c-SD	Q4:Q1	HbA1c measurement: at least five times	4.4	ICD codes evidenced HF hospitalization	NR	Age, sex, duration of diabetes, body weight, smoking, HbA1c, SBP, DBP, TC, HDL, LDL, triglycerides, albuminuria, eGFR, retinopathy, and concurrent medications
Wang 2023 C1	China	PC	Community population	98,554	53.6	78.8	4.5	FPG-VIM, FPG-SD, and FPG-CV	Q4:Q1	FPG measurement: at least three times	6.3	Clinically diagnosed HF according to the ESF HF guideline	1,218	Age, sex, DM, hypertension, baseline FPG, resting HR, LDL-C and HDL-C, SBP, DBP, BMI, hs-CRP, smoking, alcohol abuse, physical activity, and concurrent medications
Wang 2023 C2	Hongkong (China)	RC	General adult population	22,217	64.9	42.7	0.2	FPG-VIM, FPG-SD, and FPG-CV	Q4:Q1	FPG measurement: at least three times	16.2	ICD codes evidenced HF diagnosed in outpatient clinic or hospitalization	4,041	Age, sex, HbA1c, LDL-C, HDL-C, history of hypertension, DM, SBP, DBP, and concurrent medications
Manosroi 2023	Thailand	PC	Patients with prediabetes or T2DM	3,811	64.7	46.6	NR	HbA1c-SD	Q4:Q1	HbA1c measurement: at least three times	4.5	HF hospitalization evidenced by medical records	110	Age, sex, educational level, BMI, established ASCVD status, SBP, smoking status, mean HbA1c during follow-up, lipid profiles, SCr, number of HbA1c measurements, and concurrent medications

**Notes.**

AF atrial fibrillation ASCVD atherosclerotic cardiovascular disease ASV average successive variability BMI body mass index BP blood pressure CAD coronary artery disease CCI Charlson comorbidity index COPD chronic obstructive pulmonary disease CV coefficient of variation CVD cardiovascular disease DBP diastolic blood pressure DM diabetes mellitus eGFR estimated glomerular filtration rate ESF European Society of Cardiology FPG fasting plasma glucose HbA1c hemoglobin A1c HDL high-density lipoprotein HF heart failure HFpEF heart failure with preserved ejection fraction HR heart rate HTN hypertension ICD International Classification of Diseases IHD ischemic heart disease LAD left atrial diameter LDL low-density lipoprotein LVMI left ventricular mass index NR not reported PC prospective cohort RC retrospective cohort SBP systolic blood pressure SCr serum creatinine SD standard deviation T2DM type 2 diabetes mellitus TC total cholesterol TG triglycerides VIM variability independent of the mean

(Ceriello et al., 2022; Lin et al., 2021; Manosroi et al., 2023; Wan et al., 2020; Wang et al., 2023; Gu et al., 2018; Kwon et al., 2019; Li et al., 2020; Segar et al., 2020; Kaze et al., 2020).

**Table 2:** Study quality evaluation *via* the Newcastle–Ottawa Scale.

Study	Representativeness of the exposed cohort	Selection of the non-exposed cohort	Ascertainment of exposure	Outcome not present at baseline	Control for age and sex	Control for other confounding factors	Assessment of outcome	Enough long follow-up duration	Adequacy of follow-up of cohorts	Total
Gu 2018	0	1	1	1	1	1	1	1	1	8
Kwon 2019	0	1	1	1	1	1	0	1	1	7
Kaze 2020	0	1	1	1	1	1	0	1	1	7
Segar 2020	0	1	1	1	1	1	0	1	1	7
Li 2020	0	1	1	1	1	1	0	1	1	7
Wan 2020	1	1	1	1	1	1	0	1	1	8
Lin 2021	0	1	1	1	1	1	0	1	1	7
Ceriello 2022	0	1	1	1	1	1	0	0	1	6
Wang 2023 C1	1	1	1	1	1	1	1	1	1	9
Wang 2023 C2	0	1	1	1	1	1	0	1	1	7
Manosroi 2023	1	1	1	1	1	1	0	0	1	7

**Notes.**

Ceriello et al. (2022); Gu et al. (2018); Kaze et al. (2020); Kwon et al. (2019); Li et al. (2020); Lin et al. (2021); Manosroi et al. (2023); Segar et al. (2020); Wan et al. (2020); Wang et al. (2023).

### Long-term GV and HF risk

**Figure 2:**
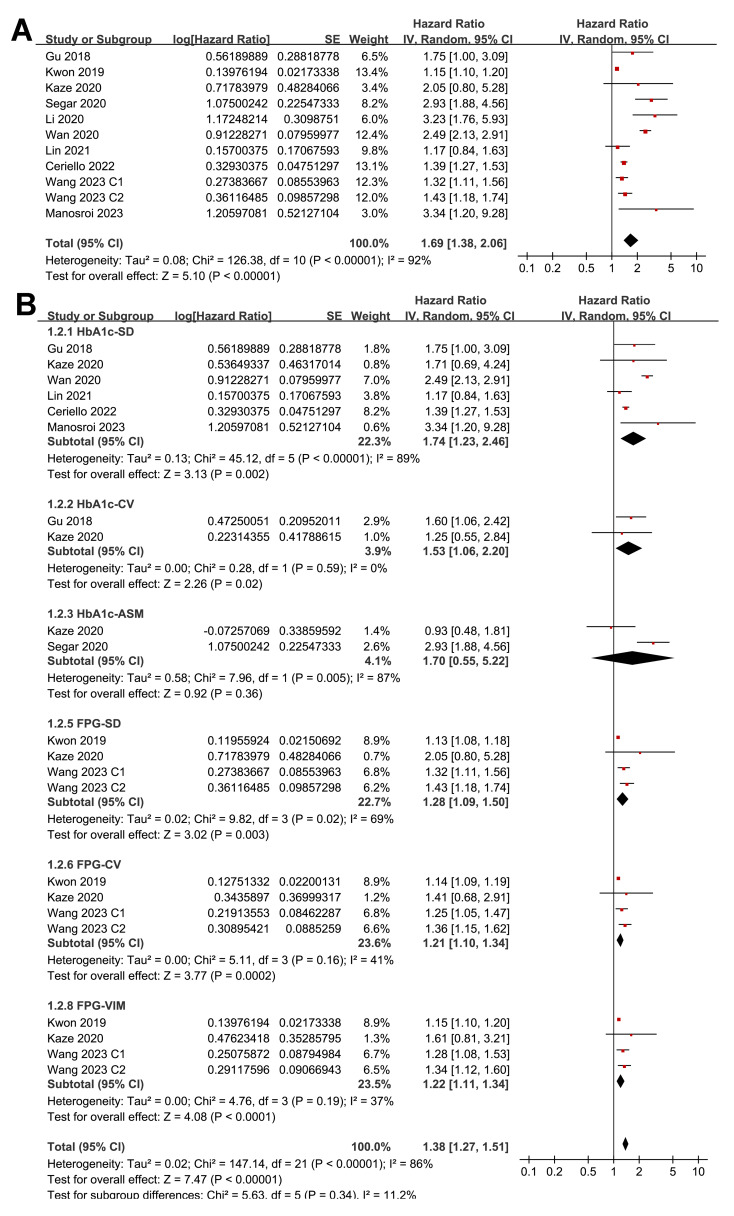
Forest plots for the meta-analysis of the association between long-term GV and the risk of HF in adult population. (A) Overall meta-analysis. (B) Subgroup analysis according to the parameters used for evaluating long-term GV (Ceriello et al., 2022; Gu et al., 2018; Kaze et al., 2020; Kwon et al., 2019; Li et al., 2020; Lin et al., 2021; Manosroi et al., 2023; Segar et al., 2020; Wan et al., 2020; Wang et al., 2023).

**Figure 3:**
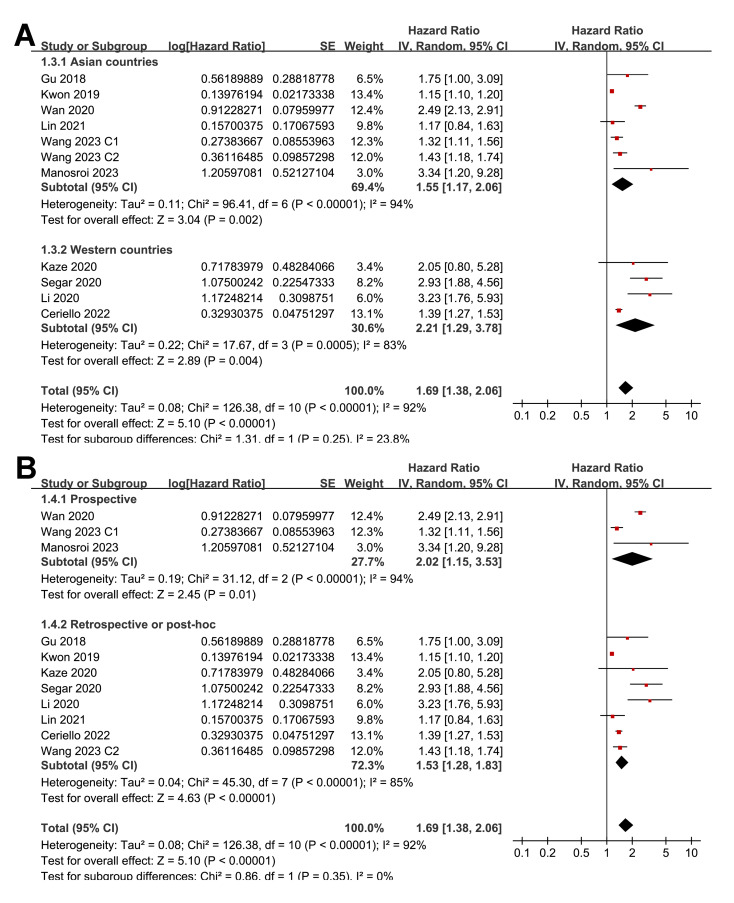
Forest plots for the subgroup analyses of the association between long-term GV and the risk of HF in adults. (A) subgroup analysis according to the study country. (B) subgroup analysis according to the study design (Ceriello et al., 2022; Gu et al., 2018; Kaze et al., 2020; Kwon et al., 2019; Li et al., 2020; Lin et al., 2021; Manosroi et al., 2023; Segar et al., 2020; Wan et al., 2020; Wang et al., 2023).

**Figure 4:**
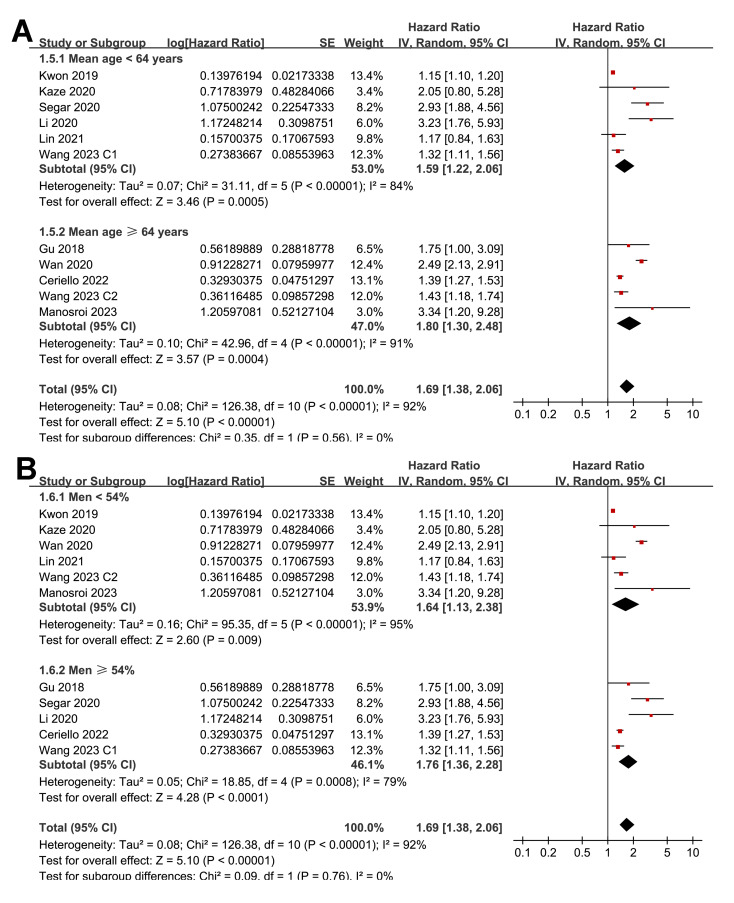
Forest plots for the subgroup analyses of the association between long-term GV and the risk of HF in adults. (A) subgroup analysis according to the mean age of the participants. (B) subgroup analysis according to the proportion of men in the population (Ceriello et al., 2022; Gu et al., 2018; Kaze et al., 2020; Kwon et al., 2019; Li et al., 2020; Lin et al., 2021; Manosroi et al., 2023; Segar et al., 2020; Wan et al., 2020; Wang et al., 2023).

**Figure 5:**
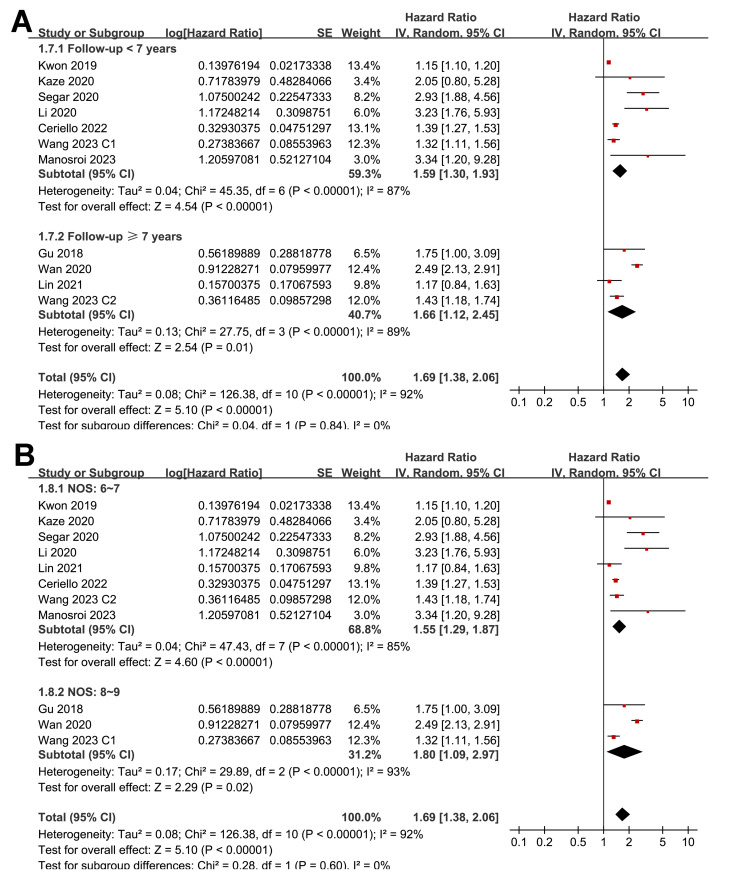
Forest plots for the subgroup analyses of the association between long-term GV and the risk of HF in adults. (A) subgroup analysis according to the mean follow-up duration. (B) subgroup analysis according to the NOS score (Ceriello et al., 2022; Gu et al., 2018; Kaze et al., 2020; Kwon et al., 2019; Li et al., 2020; Lin et al., 2021; Manosroi et al., 2023; Segar et al., 2020; Wan et al., 2020; Wang et al., 2023).

Pooled results from 11 cohorts in 10 studies (Ceriello et al., 2022; Gu et al., 2018; Kaze et al., 2020; Kwon et al., 2019; Li et al., 2020; Lin et al., 2021; Manosroi et al., 2023; Segar et al., 2020; Wan et al., 2020; Wang et al., 2023) revealed that overall, high long-term GV was associated with an increased risk of HF during follow-up (HR = 1.69, 95% CI [1.38–2.06], *p* < 0.001; *I*^2^ = 92%; Fig. 2A). Sensitivity analysis, excluding one study at a time, found no significant impact of any individual study on the results (HR = 1.51–1.80, all *p* < 0.05). Specifically, the sensitivity analysis limited to studies including patients with T2DM (Ceriello et al., 2022; Gu et al., 2018; Kaze et al., 2020; Li et al., 2020; Lin et al., 2021; Segar et al., 2020; Wan et al., 2020) revealed similar results (HR = 1.96, 95% CI [1.42–2.71], *p* < 0.001; *I*^2^ = 89%). Further sensitivity analyses also demonstrated consistent results in studies with good quality (Gu et al., 2018; Kaze et al., 2020; Kwon et al., 2019; Li et al., 2020; Lin et al., 2021; Manosroi et al., 2023; Segar et al., 2020; Wan et al., 2020; Wang et al., 2023) (NOS ≥ 7; HR = 1.78, 95% CI [1.37–2.32], *p* < 0.001; *I*^2^ = 93%), and in studies adjusting for HbA1c levels (Ceriello et al., 2022; Gu et al., 2018; Kaze et al., 2020; Manosroi et al., 2023; Segar et al., 2020; Wan et al., 2020; Wang et al., 2023) (HR = 1.95, 95% CI [1.46–2.61], *p* < 0.001; *I*^2^ = 88%). In subgroup analyses stratified by glycemic variability parameters, no significant difference was observed in the association with HF risk (*p* for subgroup difference = 0.34; Fig. 2B). In addition, further subgroup analyses recorded similar results in studies from Asian and Western countries (*p* for subgroup difference = 0.25; Fig. 3A), in prospective and retrospective or *post-hoc* studies (*p* for subgroup difference = 0.35; Fig. 3B), in participants with mean ages of <64 or ≥64 years (*p* for subgroup difference = 0.35; Fig. 4A), in populations with proportions of men of <54% or ≥54% (*p* for subgroup difference = 0.76; Fig. 4B), in studies with mean follow-up durations of <7 or ≥7 years (*p* for subgroup difference = 0.84; Fig. 5A), and in studies with the NOS scores of 6–7 and 8–9 (*p* for subgroup difference = 0.60; Fig. 5B).

### Publication bias

**Figure 6:**
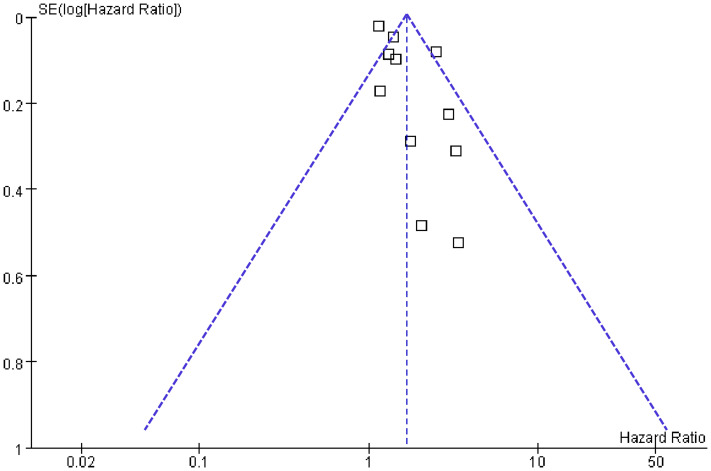
Funnel plots for estimating the potential publication bias underlying the meta-analyses of the associations between long-term GV and the risk of HF in adults.

Figure 6 displays the funnel plots evaluating the publication bias underlying the meta-analysis of the association between long-term GV and the risk of HF. The funnel plots were symmetrical on visual inspection, suggesting a low risk of publication bias. The findings were further supported by Egger’s regression analysis, which also did not suggest significant publication bias (*p* = 0.29).

## Discussion

In this meta-analysis of 11 cohorts from 10 studies involving more than 4.2 million adults, we found that high long-term GV, as measured by fluctuations in HbA1c or FPG, was significantly associated with an increased risk of HF. The pooled HR indicated a 69% higher risk of HF in individuals with high long-term GV than in those with low GV, independent of traditional risk factors. This association remained robust across various sensitivity analyses, including studies restricted to patients with T2DM, studies with high methodological quality, and studies adjusting for mean HbA1c levels. Subgroup analyses further demonstrated consistent results across study designs, populations, GV measurement parameters, geographic regions, and follow-up durations, supporting the robustness and generalizability of the findings.

Several biological mechanisms might underlie the relationship between long-term GV and the development of HF. Chronic glucose fluctuations are known to promote oxidative stress (Chang et al., 2012), endothelial dysfunction (Zhang et al., 2014), and inflammatory responses (Quincozes-Santos et al., 2017), all of which contribute to cardiovascular damage. Repetitive glycemic excursions can induce myocardial fibrosis, impair myocardial energy metabolism, and accelerate left ventricular remodeling, thus predisposing individuals to HF (Hanajima et al., 2023; Zhang et al., 2022). Additionally, long-term GV has been associated with arterial stiffness (Zhang et al., 2021) and autonomic dysfunction (Xu et al., 2016), which might further exacerbate cardiac structural and functional impairment. These pathophysiological effects suggest that GV represents a form of metabolic stress capable of contributing to HF pathogenesis beyond the effects of sustained hyperglycemia alone (Tabesh et al., 2024). The molecular pathways underlying the association between higher long-term GV and an increased risk of HF warrant further investigation.

The sensitivity analyses provided valuable insight into the consistency of the observed association. Notably, when the analysis was limited to cohorts of individuals with T2DM, the risk of HF associated with high GV remained significant and even stronger. This suggests that GV is particularly detrimental in populations already at high risk for cardiovascular complications. Furthermore, the association persisted among studies that adjusted for mean HbA1c levels, indicating that the effect of GV is not merely a reflection of overall poor glycemic control. Similarly, limiting the analysis to studies with high NOS scores did not materially alter the findings, supporting the robustness of the results. Subgroup analyses revealed no significant effect modification by the type of GV parameter (HbA1c-based *vs.* FPG-based), geographic region, study design, age, sex, or follow-up duration, suggesting that the association between long-term GV and HF risk is consistent across diverse settings and populations.

This study had several strengths. First, it represents the most comprehensive and up-to-date synthesis of evidence on this topic, incorporating studies published up to January 2025. Second, all included studies employed longitudinal designs and multivariate models, permitting temporal assessment and control of key confounding factors. Third, our use of multiple sensitivity and subgroup analyses provided a robust evaluation of the consistency of findings across various methodological and population-level factors. The large sample size and inclusion of both diabetic and non-diabetic populations enhance the generalizability of our conclusions. Nevertheless, several limitations should be acknowledged. First, most of the included studies were retrospective cohort designs, which might have introduced selection and recall bias (Song & Chung, 2010). Second, our literature search was restricted to four major English-language databases. Although these databases, supplemented by manual reference screening, are generally adequate for systematic reviews and meta-analyses, we cannot exclude the possibility that relevant studies published in other languages or indexed in other databases (*e.g.*, CENTRAL, Scopus) were missed. This restriction might have introduced language bias. The parameters and cutoffs used to define high GV varied across studies, limiting the comparability of the results and precluding a standardized threshold for clinical application. Although our subgroup analyses revealed no significant difference in the association according to GV metrics, the absence of a universally accepted definition underscores the need for future research to establish standardized metrics and clinically meaningful cutoffs for GV (Ajjan, 2024). Although all included studies used multivariate models, residual confounding remains possible. Important covariates, such as the use of sodium–glucose cotransporter 2 (SGLT2) inhibitors, which have been demonstrated to reduce HF risk (Pasternak et al., 2019), were not uniformly reported or included in the adjustment. Moreover, several key cardioprotective medications, particularly SGLT2 inhibitors and GLP-1 receptor agonists, were not consistently reported or adjusted for across the included studies. Important socioeconomic factors, such as income and access to healthcare, were also largely unmeasured. These unaccounted variables might have contributed to residual confounding, and they should be addressed in future research. The observational nature of the included studies prevented causal inference. Additionally, data were analyzed at the study level rather than the individual patient level, which restricted our ability to assess the association according to specific subtypes of HF, such as HF with reduced ejection fraction *versus* HF with preserved ejection fraction. Most included studies did not distinguish between HF subtypes such as HF with preserved *versus* reduced ejection fraction, as only one study (Gu et al., 2018) specifically examined HF with preserved with ejection fraction. Consequently, we were unable to evaluate whether the association between long-term GV and HF risk differed by HF subtype.

We were also unable to evaluate the influence of important clinical variables such as duration of diabetes, presence of comorbidities (*e.g.*, chronic kidney disease, coronary artery disease), medication adherence, and lifestyle factors, which might influence both GV and HF risk. Substantial between-study heterogeneity was observed. We could not perform robust meta-regression to formally explore potential sources of heterogeneity, as only 10 studies were available and individual-participant data were not accessible. Variables such as diabetes prevalence were reported only at the study level, preventing their examination as continuous covariates.

From a clinical perspective, our findings highlight the potential importance of long-term GV as an independent indicator of cardiovascular vulnerability. Whereas GV is not yet a standard component of cardiovascular risk assessment, these results suggest that monitoring long-term GV could provide additional prognostic information, particularly in individuals with T2DM (Howsawi & Alem, 2024). Incorporating GV into routine monitoring using readily available longitudinal HbA1c or fasting glucose records could help identify individuals at higher risk of developing HF who could benefit from closer follow-up or earlier intervention. In addition to optimizing mean glycemic control, targeting reductions in GV through tailored therapeutic approaches (*e.g.*, use of medications with more stable glucose-lowering profiles, lifestyle interventions) might further improve cardiovascular outcomes. Integrating GV metrics into electronic health records and risk prediction models might also enhance personalized risk assessment, although prospective validation is needed. Moreover, clinicians should be aware that minimizing glycemic fluctuations—not only lowering mean HbA1c—might be important in reducing the risk of HF and other diabetes-related complications (Dandona, 2017). Future research is needed to better define clinically relevant thresholds of GV, develop standardized metrics for its measurement, and investigate whether interventions targeting GV reduction can lead to improved cardiovascular outcomes. Prospective studies with detailed clinical phenotyping and individual patient-level data will be crucial to elucidate the potential differential impact of GV on HF subtypes. Moreover, randomized controlled trials or *post-hoc* analyses of trials evaluating glucose-lowering therapies could help determine whether reducing GV translates into HF risk reduction independent of changes in mean glycemia.

## Conclusions

In conclusion, this meta-analysis demonstrated that high long-term glycemic variability is independently associated with an increased risk of incident HF in adults, regardless of the diabetic status or mean HbA1c level. These findings provide compelling evidence that long-term GV is a meaningful cardiovascular risk indicator and underscore the need for greater attention to glycemic stability in clinical practice. Further research is warranted to elucidate the mechanisms underlying this relationship and determine the clinical utility of GV in risk prediction and patient management.

##  Supplemental Information

10.7717/peerj.20401/supp-1 Supplemental Information 1 PRISMA checklist

10.7717/peerj.20401/supp-2 Supplemental Information 2 The raw data extracted from the cited literature

10.7717/peerj.20401/supp-3 Supplemental Information 3 Audience

## Abbreviations

CI: confidence interval
GV: glycemic variability
HbA1c: hemoglobin A1c
HF: heart failure
HR: hazard ratio
NOS: Newcastle–Ottawa Scale
PRISMA: Preferred Reporting Items for Systematic Reviews and Meta-Analyses
PROSPERO: International Prospective Register of Systematic Reviews
SD: standard deviation
VIM: variability independent of the mean

## Additional Information and Declarations

### Data Availability

The following information was supplied regarding data availability: The raw data are available in the Supplemental File.
