# Supplementary material for: Long-term glycemic variability and the risk of heart failure: a meta-analysis
Source: PeerJ. 2025 Nov 27;13:e20401. doi: 10.7717/peerj.20401 (PMC12665266; doi:10.7717/peerj.20401)
Supplement: Supplemental Information 3 [file peerj-13-20401-s003.docx]

This systematic review and meta-analysis is primarily intended for clinicians, researchers, and healthcare professionals involved in the management of cardiovascular and metabolic diseases. Specifically, the findings are relevant to cardiologists, endocrinologists, internists, diabetologists, and primary care physicians who are engaged in the prevention, diagnosis, and treatment of heart failure and type 2 diabetes mellitus. Additionally, this work is designed to inform epidemiologists and clinical researchers focused on cardiovascular risk stratification and glycemic control, as well as policymakers and guideline developers interested in incorporating glycemic variability into cardiovascular risk assessment frameworks. By synthesizing current evidence on the association between long-term glycemic variability and heart failure risk, the study aims to support evidence-based clinical decision-making and promote further research in this emerging area.
